# A narrative review of pox: smallpox vs monkeypox

**DOI:** 10.1186/s43162-022-00174-0

**Published:** 2022-12-13

**Authors:** Samiksha Jayswal, Jagdish Kakadiya

**Affiliations:** Department of Pharmacology, Parul Institute of Pharmacy and Research, Parul University, Vadodara, 391760 Gujarat India

**Keywords:** Poxvirus, Monkeypox, Smallpox

## Abstract

The famed variola (smallpox) virus and the monkeypox virus (MPXV) are closely related, and MPXV causes a milder form of febrile rash disease in people. Human monkeypox was mostly an uncommon zoonotic illness that was restricted to West and Central African wooded areas in the twentieth century. The global population’s smallpox vaccine-induced immunity, however, has started to decrease as the number of cases and the geographic breadth have both increased significantly in this century. Several countries have seen human monkeypox outbreaks since May 2022. A possible shift in the monkeypox transmission pattern that might represent a bigger worldwide danger is raised by the atypical nature of these outbreaks, which are characterized by their high-case numbers and absence of ties to endemic countries. Here, we explore a wide range of MPXV biology topics.

## Introduction

The family of orthopoxviruses includes both monkeypox and smallpox. Animals and humans can get the DNA virus genus known as “orthopoxviruses.” Smallpox-causing variola virus is the most well-known member. The endemic monkeypox virus has produced several outbreaks in portions of Africa. Other members include the ectromelias, horsepox, raccoonpox, skunkpox, taterapox, and volepox viruses [1].

There are presently more than 70 nations where monkeypox is present,and it mostly affects tropical rainforest areas of western as well as central Africa. Six cases of monkeypox have so far been found in India. The orthopoxvirus family’s variola virus, which causes smallpox, is an acute infectious illness. Before it was eradicated, it was one of the most terrible illnesses known to mankind, responsible for millions of fatalities. It is said to have been around for at least 3000 years. Edward Jenner invented the smallpox vaccine in 1796, which was the first effective vaccination ever. He noticed that milkmaids who had previously had cowpox did not contract smallpox and demonstrated how other individuals may be protected against smallpox with a similar vaccination.

In 1967, the World Health Organization began a more aggressive campaign to eliminate smallpox. For many years, widespread vaccination and surveillance were carried out all throughout the world. The final natural instance was recorded in 1977 in Somalia. Smallpox was the first infectious illness to acquire this distinction when it was proclaimed eliminated by the WHO in 1980 (https://www.who.int/groups/who-advisory-committee-on-variola-virus-research/meeting-documents).

The monkeypox virus was first found in the year 1958 [2, 3]. The vaccinia vaccine formerly provided coincidental immunity against the monkeypox virus, but the eradication of smallpox and the consequent absence of immunization campaigns allowed monkeypox to become clinically relevant [4]. Furthermore, given that the majority of monkeypox cases in Africa occur in rural areas, it is possible that underreporting is occurring, which might result in an underestimate of the pathogen’s potential harm [5].

### History

The current outbreak of monkeypox has been related to the eradication of smallpox and the end of routine immunization. Getting vaccinated against smallpox resulted in some cross-protection against monkeypox. Most people no longer have immunity against both smallpox and monkeypox since smallpox was removed from the list of recommended immunizations in nations like the USA in 1972.

Around 1000 B.C., areas like India and Egypt experienced the first endemic spread of smallpox. Up to 30% of those who contracted the disease died as a result of its worldwide trade-based spread. Those who managed to survive the excruciating blisters frequently developed scars, disfigurement, or blindness.

Countries like the USA had almost completely eradicated smallpox in the 1940s. However, there were still 10–15 million cases worldwide in 1967, which indicated that the illness was still killing millions of people. Smallpox eradication was the objective of a vaccine program started by international health authorities.

The virus had killed more people over the course of three millennia by the time health officials pronounced it dead in October 1979 than The Plague or any other sickness. And the danger is still present.

In 1988, a piece in the International Journal of Epidemiology cautioned that monkeypox outbreaks will become more common and severe due to declining smallpox immunity. However, they did observe that compared to smallpox, monkeypox was far less fatal and contagious.

In the 1990s, there were 520 confirmed cases of monkeypox in Central Africa. Between 2010 and 2019, there were approximately 18,000 suspected cases. Monkeypox should not be taken lightly, according to a paper that was published in PLOS Neglected Tropical Diseases in 2022.

### Understanding the monkeypox disease

Along with cowpox, smallpox, camelpox, and varicella, monkeypox is considered a member of the orthopoxviral family (aka chickenpox). The diagnosis of monkeypox frequently requires laboratory confirmation. Patients with monkeypox show symptoms that are comparable to those of smallpox. Both infections have an incubation period of up to 17 days. The person could subsequently feel uncomfortable all over, have a fever, and have a headache. Hard lesions that eventually fall off the body grow over time.

Monkeypox, unlike smallpox, usually does not result in death. Additionally, it is difficult to spread between people, especially when compared to influenza and COVID-19, two respiratory illnesses that are extremely infectious. Monkeypox spreads when a person touches a lesion on an infected individual, which might occur during intimate interactions. Another route for respiratory transmission, according to the CDC, is “prolonged face-to-face contact” (https://www.discovermagazine.com/health/the-history-of-smallpox-disease-and-why-it-relates-to-monkeypox).

### Etiology

The orthopoxviral genus and species names for the monkeypox virus include Poxviridae, chordopoxvirinae, and orthopoxvirus family.

The monkeypox virus is rather large when seen under an electron microscope (200–250 nanometers). A lipoprotein sheath surrounds the concrete block dual DNA genome of a xenovirus. Poxviruses depend on the host ribosomes for mRNA translation, but they also have all of the necessary replication, transcription, assembly, and exit proteins in their genome [6–8].

### Epidemiology

Although contaminated clothes or bedding can potentially spread the infection, respiratory droplet nuclei are the main mode of smallpox transmission [9, 10].

Despite the fact that smallpox is less communicable than measles, chickenpox, or influenza, subsequent incident rates among uncontrolled contacts range from 37 to 66%. Patients are most contagious from the beginning of the enanthem through the first 8 to 12 days of rash [11–13]. Only family members or healthcare professionals are often impacted by secondary incidents. Patients who are coughing or who are sickly can spread several viruses. One patient in Meschede, Germany, transferred smallpox to 17 patients throughout three hospital floors during the incubation period. This large-scale outbreak was linked to the cough of the patient as well as the facility’s less relative humidity and air currents.

Due to the possibility that aerosolized orthopoxviruses can survive longer in cooler temperatures and lower humidity levels, smallpox cases are more prevalent in the winter and early spring [14, 15]. Since so few Americans under the age of 30 have taken the vaccination, everyone in the country is susceptible to smallpox. Born before 1972, those who had immunizations may still be somewhat protected; if exposed, they may suffer a milder sickness and be less likely to pass it to others.

In 2022, a global monkeypox outbreak started. Men’s sexual or close intimate contact was reported by 94% of male monkeypox patients in the USA who had data on their cases; racial and cultural minority groups appear to be disproportionately affected. Different clinical symptoms from the typical monkeypox, with more people having genital rashes than prodrome (https://www.cdc.gov/mmwr/volumes/71/wr/mm7132e3.htm#:~:text=A%20global%20monkeypox%20outbreak%20began,appear%20to%20be%20disproportionately%20affected).

### Transmission

Prior to smallpox’s eradication, smallpox has been mostly transmitted by prolonged, direct face-to-face contact. Smallpox victim’s mouth and throat blisters originally formed and then spread infection (early rash stage). They transmitted the virus by sneezing, coughing, or allowing their mouth or nose drops to come into proximity with others. Up until the final patch of their smallpox had come off, they were dangerous. It was also present in the patient’s wounds. These materials or the items they contaminate, such as bedding or clothes, can allow the infection to spread. When washing the bedding or clothing of smallpox victims, caregivers had to use gloves and take precautions to avoid contracting the disease.

Rarely has smallpox spread by air in confined spaces like a building (airborne route). Only people can spread smallpox. There is no proof that animals or insects may spread smallpox, according to scientists (https://www.cdc.gov/smallpox/transmission/index.html).

Anyone can contract monkeypox through close, direct, and frequent contact with the skin, which includes the following:

- Contacting surfaces, materials (such as towels, clothes, or bedding), and things that have been touched by someone who has monkeypox
- Coming into contact with nasal secretions

During close touch, this direct contact may occur, including the following:

- ➣ Touching the genitals, or anus of a person who has monkeypox, or having oral sex, or non-penetrative and penetrative activities with them (butthole)
- ➣ Cuddles, kissing, and body massages
- ➣ Prolonged face exchanges
- ➣ Handling objects and fabrics

A person with monkeypox can spread it to other people from the time symptoms start until the rash has completely recovered and a new layer of skin has formed. The illness typically lasts14–28 days (https://www.cdc.gov/poxvirus/monkeypox/if-sick/transmission.html).

### Clinical signs and symptoms

It takes 7 to 14 days for smallpox to incubate. The temperature frequently increases beyond 40°C before dropping over the course of 2 to 3 days. A day before the rash appears, there is an enanthem throughout the mouth. The rash starts as a small, red macule which over the time of 3–4 days develops into papules with a diameter of 3 to 4 mm. After 4–5 days, the papules then develop into vesicles with a diameter of 2 to 5 mm. Lesions start from the face and extremities, but they spread.

Five to 7 days after the start of the rash, 4 to 6 mm-diameter pustules appear and last for 5 to 8 days before umbilication and crusting. Five to 8 days following the appearance of the rash, there may be a second, less obvious fever increase, mainly if the patient has any other kind of bacterial infection. After 14 days of the eruption, the crusts start to separate. Smallpox lesions are often all in a similar state of development. The most persistent lesions are those on the palms and soles. Toxemia, linked to immunological complexes, and hypotension are blamed for smallpox death. Sixty-five to 80% of people with severe smallpox get pockmarks or pitted lesions [9].

The vast sebaceous glands on the face have the propensity to become infected; therefore, these lesions are frequent there. About 1% of individuals may develop pan ophthalmitis, which can lead to blindness from viral keratitis or subsequent eye infections. Up to 2% of children with smallpox go on to develop arthritis; infection of the developing bones is assumed to be the reason. Less than 1% of smallpox victims get encephalitis. Although cough is the uncommon symptom, the chance of respiratory problems increases with illness severity; pneumonia or bacteremia may have a high fatality rate.

Five different forms of smallpox are covered by a helpful categorization offered by WHO [9]. A study of 3500 patients in India served as the basis for the categorization. The “typical” form of smallpox, made up approximately 92% of cases in that research, with a 30% case fatality rate [10]. The milder, “modified” form made up 2% of instances among those who had never had a vaccination and 25% in those who had. Lesions in patients with the modified instances were lesser in patients with the first type, and they progressed more rapidly. These cases were seldom deadly. In 7% of cases, flat lesions that consolidated and progressed lesser than those of variola were observed; in unvaccinated individuals, the case fatality rate for the flat form was 96% [9].

Lesions in monkeypox are deep-seated, well-circumscribed, stiff or rubbery, and frequently develop umbilication.

During the ongoing worldwide pandemic:

1. The oral, vaginal, and anorectal regions are common sites for lesions
2. Rash does not necessarily spread to several locations on the body
3. Rash may just cause a few lesions or just one solitary lesion
4. Rash does not always show up on your hands or feet

In the present epidemic, rectal discomfort, or rectal bleeding has been regularly recorded.

Until they start to heal, lesions are frequently reported as painful, then they turn irritating (crusts). Other prodromal signs include fever and respiratory symptoms.

Any given area of the body may experience the simultaneous development and evolution of many lesions. Before scabbing over and desquamation, lesions go through four phases of development: macular, popular, vesicular, and pustular. The time of incubation is 3–17 days. A person may feel fine and not have any symptoms throughout this period. Usually, the disease lasts 2 to 4 weeks. The starting health of the person and the exposure route might affect the illness’s severity. Compared to the Congo Basin virus clade, Africa is the clade responsible for the present outbreak.

Variola sine eruption, the most severe form of smallpox, affects those who have already received vaccinations or newborns who have maternal antibodies. Variola sine eruption has not been shown to transmit clinical smallpox; infected people are not symptomatic or only have a brief increase in temperature, and symptoms similar to influenza (https://www.cdc.gov/mmwr/volumes/71/wr/mm7132e3.htm#:~:text=A%20global%20monkeypox%20outbreak%20began,appear%20to%20be%20disproportionately%20affected, https://www.cdc.gov/smallpox/transmission/index.html). The Americas and certain parts of Africa are the main locations affected by the mild illness variola minor. Less than 1% of those who get the illness eventually pass away (https://www.cdc.gov/poxvirus/monkeypox/if-sick/transmission.html). An infection with smallpox leads to lifelong antibodies [1].

### Complications and prognosis

#### Complications

##### Smallpox

- Bacterial superinfections of organs as well as widespread sepsis are symptoms of smallpox [1].
- Other conditions include keratitis, rheumatoid arthritis, pneumonia, encephalitis (2 in 1000 variola severe cases), face pockmarks (70–80% of survivors), blindness, and limb abnormalities

##### Monkeypox

The mode of exposure and the viral strain affect how severe a monkeypox illness is. Anyone with a history of atopic dermatitis or other exfoliative skin disorders such as eczema, burns, ringworm, herpes zoster virus infection, herpes simplex virus infection, extreme acne, drastic diaper skin infections, psoriatic arthritis, or Darier’s disease; young kids, especially those under the age of 7; pregnant women; and breastfeeding mothers are at risk for developing a serious illness.

#### Prognosis

##### Smallpox

Multiorgan failure is the main cause of death in severe sickness, and mortality is above 32% in variola major patients compared to 1 to 2% in variola moderate patients (caused by inflammatory mediators and direct viral cytopathic effects) [1, 16].

##### Monkeypox

There are two distinct clades of the monkeypox virus. The west African lineage has a brighter prognosis when the case fatality rate is less than 1%. On the other side, the middle basin clade (middle African clade) is more lethal, with a standardized mortality rate of up to 12% in young, uninfected people. Within 4 weeks following the onset of symptoms, patients often recover completely, with the potential exception of bruises and hyperpigmentation [1, 17], (https://www.who.int/groups/who-advisory-committee-on-variola-virusresearch/meeting-documents).

#### Evaluation and diagnosis

##### Smallpox clinical case definition

A condition with no other clear explanation, an acute fever of less than 102°F (37.4°C), and a rash with firm, deep vesicles or pustules at the same stage of development [18].

##### Patient evaluation method

Numerous rash conditions can cause vesicles and pustules. Smallpox is unlikely to affect a patient with a rash illness, but it is still possible. The algorithm “Assessing Sick people for Smallpox: Acute, Generalized Vesicular or Pustular Skin infection Illness Protocol” offers a uniform method for assessing patients with acute, severe vesicular, or pustular rash illness by offering clinical cues for differentiating smallpox from varicella and other rash illnesses.

If a patient exhibits an acute, widespread vesicular or pustular rash, use the algorithm and the recommendations below to determine whether smallpox is the cause. A risk analysis from the methodology will assist in determining the best course of action for medical treatment and population health. Consult the state or local community health department for advice. The CDC can be contacted by state and local public health organizations at 770-488-7100 for guidance about elevated individuals or other difficult situations [18, 19].

##### Protection against infection measures

- Move the patient into a room with isolation for airborne infections (AIIR). If a private room is not available, use one. No one should be left waiting in a public location.
- Notify the infection control department (if in a healthcare facility).
- Implement the appropriate standard, contact, and airborne safety precautions. N95 respirators, gloves, and appropriately sized gowns are required for both personnel and visitors.
- If the patient needs to be transported, cover their rash with a sheet and their mouth, and nose with a N95 respirator or surgical mask [18].

##### Physical examination as well as a history

Ask your patient-specific questions regarding any symptoms present 1 to 4 days before the start of the rash, including prodromal symptoms and clinical features, interaction with any sick persons (particularly those with a rash illness), recent travel experiences, and other information. A history of herpes zoster or varicella, exposure to odd or ill animals, a medical history involving prescription medication, and vaccination history for varicella (vaccine available since 1995) discovers the risk category. The major and minor diagnostic criteria for the illness should be used to categories the patient’s risk of developing smallpox. Contact your local or state health agency with any questions or if you need assistance identifying the risk category for your patient. Furthermore, CDC may be called at 770-488-7100 for advice [18].

#### Important smallpox diagnostic standards

- There is a febrile prodrome that occurs 1 to 4 days before the appearance of the rash: A temperature of 101°F (37.3°C) OR at least one of the following:
- Backache, chills, nausea, vomiting, and severe stomach ache.
- Classic smallpox lesions have deep-seated, firm/hard, round, well-circumscribed vesicles, or pustules. As a lesion grows, it could become umbilicated or confluent.
- Lesions that are all in the same stage of development on ONE area of the body.
- The face and distal extremities contain the largest concentration of lesions in the centrifugal distribution of the rash, which is a diagnostic feature of minor smallpox.
- The face, biceps, or oral mucosa/palate as the primary areas of infection severity: the patient appears to be dead or poisoned.
- Lesions slowly progressed from macules to papules to pustules over the course of days in a rash (each stage lasts 2 to 3 days)
- Ailments on the soles or palms [18–20].

##### Monkeypox

Include monkeypox in the differential diagnosis as well. The main difference between smallpox and monkeypox is that smallpox does not cause lymphadenopathy, but monkeypox causes swelling in the lymph nodes. Lymph node swelling may only impact a few localized areas of the body or it may affect several different locations (e.g., neck and armpit). Consult the client [19].

#### Treatment

##### Smallpox

Patients with smallpox are often treated with supportive treatment. If administered within 2 to 3 days of the initial exposure, vaccination with replication-competent smallpox vaccines (such as ACAM2000 and APSV) can prevent or minimize the severity of the illness. If used within the first week of exposure, they could lessen symptoms.

Isolation and adherence to appropriate infection and environmental controls are required while treating smallpox patients in a medical setting [18–20].

#### Antivirals

##### Tecovirimat

The first medication with an indication for the treatment of smallpox, tecovirimat (also known as ST-246 or by its brand name Pox), was authorized by the U.S. Food and Drug Administration (FDA) in July 2018. Although there are little effectiveness data in humans, tecovirimat has been utilized in the treatment of severe adverse reactions following immunization with the vaccinia virus. With the aid of related orthopoxviruses and variola, in vitro investigations were used to demonstrate tecovirimat’s efficacy against smallpox. Numerous animal model studies comparing survival in animals infected with either the variola virus or other closely related Orth poxviruses have also shown the effectiveness of tecovirimat therapy. Additionally, administering tecovirimat to prairie dogs exposed to the monkeypox virus reduced mortality and the appearance of morbidity. Tecovirimat’s safety was evaluated in 359 healthy human volunteers.

Tecovirimat was approved under the FDA’s animal rule, which allows efficacy findings from adequate and well-controlled animal studies to support an FDA approval when it is not feasible or ethical to conduct efficacy trials in humans. The FDA Antimicrobial Drugs Advisory Committee voted unanimously (17 to 0) that the benefits of tecovirimat treatment for smallpox.

##### Brincidofovir

The FDA authorized brincidofovir, also known by the trade name TEMBEXA, for the treatment of smallpox in June 2021. Studies conducted in vitro with variola and orthopoxviruses linked to smallpox demonstrated brincidofovir’s efficacy against the disease. Numerous research using animal models assessing the survival of animals infected with either the variola virus or other closely related orthopoxviruses have shown the effectiveness of brincidofovir therapy. Bincidofovir’s safety was examined for non-smallpox purposes, especially in recipients of bone marrow transplants. The FDA’s animal rule permitted the FDA to approve brincidofovir [18, 19].

##### Cidofovir

Cidofovir has been demonstrated in laboratory studies to be effective against the virus that causes smallpox and to cure animals that had conditions comparable to smallpox. Smallpox patients have not been tested for cidofovir; however, healthy individuals and those suffering from other viral infections have. Ongoing testing is done to determine the toxicity and efficacy

of this medication. It is not FDA-approved for the treatment of variola virus infections (smallpox), but with the right regulatory framework, it may be used in an epidemic (such as an investigational new drug [IND] protocol or Emergency Use Authorization) [1].

##### Monkeypox

There are no recognized, efficient treatments for monkeypox infection at this time. Supportive symptom management is the approach used to treat viral infections. But there are steps that may be taken to prevent an outbreak. The infected individual has to be kept apart, covered as much as they can with a surgical mask, and separated. The infected individual should remain isolated, wear a surgical mask, and keep lesions covered as much as is practicable until all lesion crusts have naturally fallen off and a new skin layer has developed. In extreme circumstances, compounds that have been shown to be effective against orthopoxviruses in animal testing and against the severe side effects of the vaccinia vaccine may be studied.

Contact between wounded skin or mucous membranes with an infected patient’s body fluids, respiratory droplets, or scabs is a “high-risk” exposure that demands post-exposure immunization as soon as feasible. According to the CDC, vaccination after 4 days of exposure may prevent the disease from developing, and vaccination within 15 days may lower the disease’s severity. The altered vaccine has issues with replication. The Ankara vaccine is given in a two-shot series separated by four weeks, and it has a superior safety profile than the first- and second-generation smallpox vaccines. In contrast to live vaccinia virus preparations, administering modified vaccinia, Ankara, does not result in a skin lesion or raise the risk of local or widespread transmission [18]. Clinical trials have shown that modified vaccinia Ankara is secure and effective. Additionally, clinical studies have demonstrated that modified vaccinia Ankara is safe and promotes the formation of antibodies in individuals with atopy and weakened immune systems—conditions that are known to be contraindicated for the injection of live vaccinia [18–21].

## Conclusion

This review provides readers with information on the current situation surrounding smallpox and monkeypox and presents the state of the art in terms of the impact on public health, epidemiology, clinical characteristics, diagnosis, management, therapy, and prevention. The research on this subject is scarce despite the fact that the virus is not new and has frequently caused outbreaks in less developed and rural parts of Africa. It is the ideal illustration of the potentially explosive combination of manmade causes and zoonotic spillover that accounts for the vast majority of the potential for epidemics throughout the planet. Smallpox and monkeypox may be treated and prevented, unlike other neglected illnesses, but access is difficult in the region of the globe where they are most prevalent.

Responding to continuing outbreaks where and when they occur rather than waiting for them to move elsewhere is necessary for preparing for epidemics and global health concerns. However, it will be crucial that each step in scientific advancement and medical innovation can also be accessible to those who can benefit most.

Regarding the ongoing multi-country epidemic, there are still significant issues and unanswered problems, such as the abnormally rapid emergence of cases across multiple nations, the unpredictability of the disease’s future course, and the dearth of effective medical countermeasures. Furthermore, we promote the use of vaccinations as soon as feasible, at least for particular demographic groups and healthcare professionals, because smallpox infection immunizations protect against MPX and the vaccine is ready. Finally, this epidemic is reawakening the syntomic risk spectra that the recent and continuing COVID pandemic has conjured. Therefore, it is essential to step up research efforts to reduce the infectious risk throughout the long run as well as in the near term and for all countries of the world.

## Data Availability

Not applicable.

## References

[1] COgden HG (1987) CDC and the Smallpox Crusade. US Department of Health and Human Services, Public Health Service, Centers for Disease Control

[2] Cho CT, Wenner HA (1973) Monkeypox virus. Bacteriol Rev 37(1):1–18 10.1128/br.37.1.1-18.1973 PMC413801 4349404

[3] Ladnyj ID, Ziegler P, Kima E (1972). A human infection caused by monkeypox virus in Basankusu Territory, Democratic Republic of the Congo. Bull World Health Organ..

[4] Nguyen PY, Ajisegiri WS, Costantino V, Chughtai AA, MacIntyre CR (2021). Reemergence of human monkeypox and declining population immunity in the context of urbanization, Nigeria, 2017-2020. Emerg Infect Dis.

[5] Sklenovská N, Van Ranst M (2018). Emergence of monkeypox as the most important orthopoxvirus infection in humans. Front Public Health..

[6] Voigt EA, Kennedy RB, Poland GA (2016). Defending against smallpox: a focus on vaccines. Expert Rev Vaccines.

[7] Walsh D (2017). Poxviruses: slipping and sliding through transcription and translation. PLoS Pathog..

[8] Alakunle E, Moens U, Nchinda G, Okeke MI (2020). Monkeypox virus in Nigeria: infection biology, epidemiology, and evolution. Viruses..

[9] Fenner F, Henderson DA, Arita I, Jezek Z, Ladnyi ID (1988). Smallpox and its eradication.

[10] Dixon CW (1962). Smallpox.

[11] Hope Simpson RE (1952). Infectiousness of communicable diseases in the household (measles, chickenpox, and mumps). Lancet.

[12] Mack TM, Thomas DB, Muzaffar KM (1972). Epidemiology of smallpox in West Pakistan. II. Determinants of intravillage spread other than acquired immunity. Am J Epidemiol.

[13] Wehrle PF, Posch J, Richter KH, Henderson DA (1970). An airborne outbreak of smallpox in a German hospital and its significance with respect to other recent outbreaks in Europe. Bull World Health Organ.

[14] Harper GJ (1961). Airborne micro-organisms: survival tests with four viruses. J Hyg (Lond).

[15] Huq F (1976). Effect of temperature and relative humidity on variola virus in crusts. Bull World Health Organ.

[16] Moore ZS, Seward JF, Lane JM (2006). Smallpox. Lancet.

[17] Pastula DM, Tyler KL (2022) An overview of monkeypox virus and its neuroinvasive potential. Ann Neurol 92(4):527–31. 10.1002/ana.26473 35932225

[18] McCollum AM, Damon IK (2014). Human monkeypox. Clin Infect Dis..

[19] Petersen BW, Kabamba J, McCollum AM, Lushima RS, Wemakoy EO, Muyembe Tamfum JJ, Nguete B, Hughes CM, Monroe BP, Reynolds MG (2019). Vaccinating against monkeypox in the Democratic Republic of the Congo. Antiviral Res..

[20] Reynolds MG, McCollum AM, Nguete B, Shongo Lushima R, Petersen BW (2017). Improving the care and treatment of monkeypox patients in low-resource settings: applying evidence from contemporary biomedical and smallpox biodefense research. Viruses..

[21] Baker RO, Bray M, Huggins JW (2003) Potential antiviral therapeutics for smallpox, monkeypox and other orthopoxvirus infections. Antivir Res 57(1-2):13–23 10.1016/S0166-3542(02)00196-1 PMC9533837 12615299

